# Metrorrhagia; Hemorrhage from the Womb

**Published:** 1878-01-20

**Authors:** I. J. M. Goss


### *METRORRHAGIA—HEMORRHAGE FROM THE WOMB.*

By I. J. M. Goss, A.M., M.D.

This is a more or less profuse flow of blood from the uterus at any other time than that of the regular menstrual period. It may occur in the non-pregnant state as the result of fluxion to that organ, or in consequence of morbid growths in the womb, or it is frequently the result of "a change of life," as the women call it. It may occur also in pregnancy, and is then the forerunner of abortion. Hemorrhage during the second half of pregnancy is a sign of a placenta previa, frequently. It may occur after delivery, as the result of imperfect contraction of the uterus, or from large coagula within the uterus, where the contractions are imperfect. I have seen some cases from inflammatory irritation of the womb.

*Treatment.*—Many remedies have been used, but most of them have signally failed in some cases. Where the flow is continuous, and there is nausea, vomiting, prostration, fainting and palpitation of the heart, then apocynum cannabinum will often arrest the hemorrhage; it may be given in doses of 5 drops every two hours. If the hemorrhage occurs after labor, or after abortion, the oil of erigeron canadensis, in doses of 5 or 10 drops, will often arrest it in a few hours; very frequently, the first dose will arrest it. If the hemorrhage is passive from anæmia, then hamamelis, in doses of 1 to 3 drops every half hour, will generally arrest it. In cases of imperfect contraction, the tincture of nux vomica, 5 drops every four hours, will stay it.

Menorrhagia.—This is another menstrual anomaly, and nearly allied to the preceding one, but one that requires a distinct notice. It occurs at the time of the menstrual period. It may keep regular periods, or come too soon, or last too long; in some cases the flow is profuse and too early in its occurrence. Its causes are various, as structrual changes in the uterus, morbid growths, strangulation of the blood in the uterine veins, depending upon lung and heart diseases, or upon excessive sexual excitements. It may be the result of a hemorrhagic diathesis, as in scurvy, purpura, etc. In this form of hemorrhage the blood may be either fluid or coagulated, and of various colors. Stout plethoric women may endure menorrhagia for a long time; weak, feeble women soon show signs of anæmia and great prostration, which will bring on dropsy unless the hemorrhage be checked. I have a case now under treatment that resulted in diabetes mellitus, from menorrhagia in a girl.

*Treatment.*—Where the flow is profuse and too early, with dark coagula, aching of the limbs, pain in the back, down the thighs, with pressing down, nervousness, headache; then cimicifuga will generally aid in checking the flow, and correcting the nervous peculiarities in the case; it should be given in 5 drop doses, three or four times a day. At the same time the oil of erigeron may be given in 5 drop doses every two hours. If there is labor-like pains, gelseminum may be given with good effect, in doses of 10 drops, every two hours, with trillium pend., in doses of 30 drops of the saturated tincture.

Prof. Meigs was accustomed to use a solution of alum in these cases, given internally, strongly flavored with nutmeg. Dose, 15 to 20 grains.—[Ed. Rec.

